# Egg laying during stress-induced sleep of *Caenorhabditis elegans* is reduced due to behavioral quiescence and fertility defects

**DOI:** 10.17912/micropub.biology.001735

**Published:** 2025-07-15

**Authors:** Sanjita Subramanian, Nikki Diya, Matthew D. Nelson

**Affiliations:** 1 Pharmacology & Physiology, Drexel University, Philadelphia, Pennsylvania, United States; 2 Biology, Saint Joseph's University, Philadelphia, Pennsylvania, United States

## Abstract

Sleep is a reversible state, characterized by the inhibition of periodic behaviors that occur during waking hours. *Caenorhabditis elegans* demonstrates stress-induced sleep following exposure to environmental stressors, like noxious heat or ultraviolet irradiation. During this time, animals inhibit movement, feeding, and defecation, behavioral quiescence largely controlled by neuropeptide signaling from the ALA and RIS sleep interneurons. Here, we tested whether egg retention and/or production which occurs during suboptimal environmental conditions, is regulated by the ALA and/or RIS, or other neuropeptides. We find that during stress-induced sleep, worms reduce egg-laying behavior and egg production (i.e., fertility). While the behavior is modestly modified in the absence of the ALA and RIS, as well as some neuropeptides, fertility is regulated by other mechanisms.

**Figure 1. Egg laying during stress-induced sleep of Caenorhabditis elegans is reduced due to behavioral quiescence and fertility defects:**
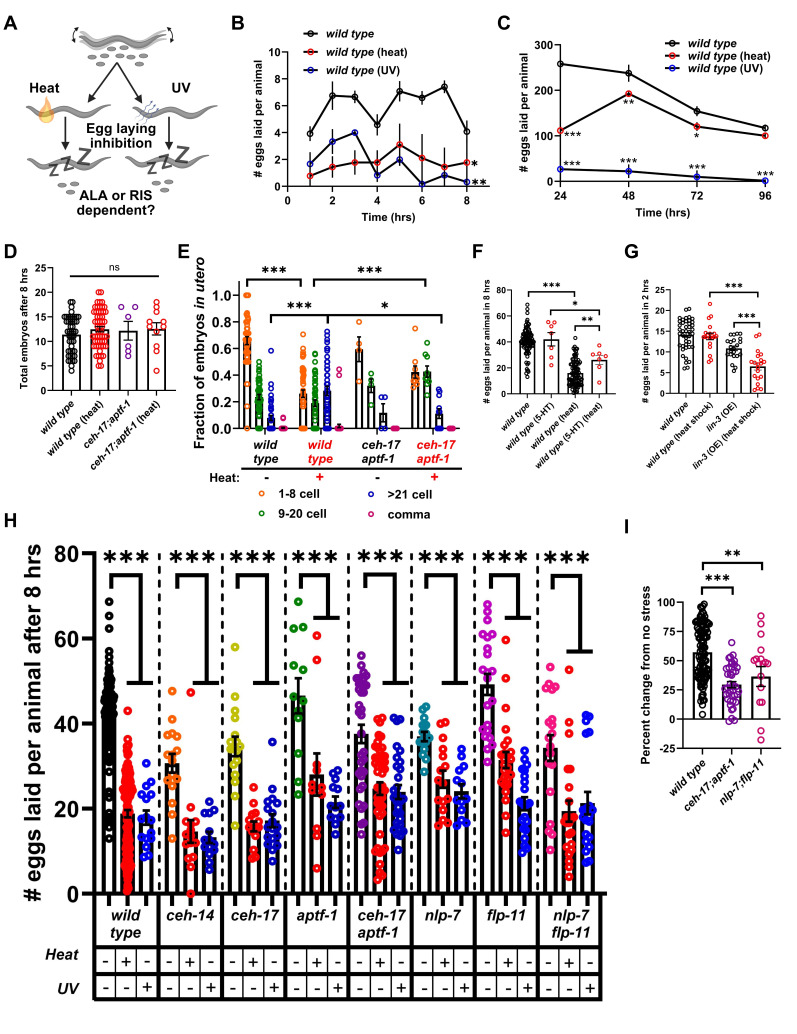
**A)** A schematic of the purpose of the study, which was to determine if the ALA and/or RIS sleep neurons are required for egg laying inhibition during stress-induced sleep. Created with BioRender.com. **B)** Average number of eggs laid per animal each hour after heat or UV stress in wild-type animals. **C)** Average number of eggs laid per animal each day after heat or UV stress in wild-type animals. For both **B** and **C** , averages at each time point were compared by two-way ANOVA followed by Sidak's multiple comparisons test (*p<0.05, **p<0.01, at each time point, average of 4 trials each with 3 animals). **D)** Total number of embryos *in utero* either 8 hours after heat stress, or in control animals that were not stressed, for wild-type (N≥47), and *ceh-17 ( np1 ); aptf-1 ( gk794 )* animals (N≥6). **E)** Average fraction of embryos in different stages of development *in utero* in controls (no stress), and 8 hours post heat stress, in wild-type and *ceh-17* ; *aptf-1* animals (N≥5). **F)** Average number of eggs laid in controls (no stress) and 8 hours after heat stress in wild-type animals exposed to 25mM serotonin (5-HT). **G)** Average number of eggs laid in wild-type and transgenic animals capable of over expressing *lin-3* from the inducible heat shock promoter. **H)** Average number of eggs laid by controls (no stress) and during heat- and UV-induced sleep in wild-type, *ceh-14* ( *ch3* ), *ceh-17* ( *np1* ), *aptf-1* ( *gk794* ), *ceh-17 ( np1 ); aptf-1 ( gk794 ), nlp-7* ( *tm2984* ), *flp-11* ( *tm2706* ) *,* and *nlp-7* ( *tm2984* ) *; flp-11* ( *tm2706* ) animals *.* **I)** Percent change between no stress and heat stress in wild-type, *ceh-17 ( np1 ); aptf-1 ( gk794 ),* and *nlp-7* ( *tm2984* ) *; flp-11* ( *tm2706* ) animals. Statistical significance for **D-I** was calculated by one-way ANOVA followed by Tukey's multiple comparisons test (*p<0.05, **0<0.01, ***p<0.001). Error bars represent standard error of the means.

## Description

*Caenorhabditis elegans* employs an oviparous reproductive strategy *,* in which rhythmic egg laying occurs during active states when resources and conditions are favorable (Schafer, 2005; Waggoner et al. 1998). Environmental stress that disrupts survival and causes tissue damage, such as hyperosmolarity, noxious mechanical stimuli, starvation, CO _2_ exposure, and extreme temperatures, lead to egg retention (Sawin, 1996; Fenk and Bono, 2015), which may allow for maternal control and enhanced survival of offspring (Mignerot et al. 2024). Stress-induced sleep occurs following exposure to environmental insults such as noxious temperature, injury, infection, ultraviolet irradiation and oxidative stress (Hill et al. 2014; DeBardeleben et al. 2017; Sinner et al. 2021; Iannacone et al. 2024). Inhibition of stress-induced sleep reduces longevity (Hill et al. 2014), so, this sleep may promote energy reallocation towards repair and recovery. During stress-induced sleep, locomotion, feeding, and defecation are reversibly inhibited; this behavoral quiescence is controlled by two sleep neuropeptidergic interneurons called ALA and RIS (Hill et al. 2014; Konietzka et al. 2020). ALA and RIS are activated during sleep by epidermal growth factors released from damaged cells (Hill et al. 2024), which leads to the the secretion of neuropeptides which inhibit wake behaviors (Nelson et al. 2014; Nath et al. 2016; Honer et al. 2020). While it is clear that egg retention occurs in response to chronic stress, a quantification of egg laying following acute stress exposure and during stress-induced sleep, and a role for the ALA and RIS neurons has not been tested ( **Figure 1A** ).

Chronic heat stress reduces fertility and egg laying behavior (McMullen et al. 2012; Plagens et al. 2021), and prolonged exposure to UV-A light decreases brood size (Prasanth et al. 2016). First, we assessed wild-type egg laying during heat- and UV-induced stress-induced sleep, which can occur following acute stress exposures. Specifically, we measured egg laying over 8 hours following acute noxious heat (30 minute exposure to 37 ^o^ C), or UV-shock (Stratagene, 1500J/m ^2^ ), both known to cause prolonged sleep (Hill et al. 2014; DeBardeleben et al. 2017). Animals exposed to heat or UV laid fewer eggs every hour for the duration of the 8 hours ( **Figure 1B** ), with controls laying ~40 eggs per worm, and heat or UV exposed animals ~25 and ~20 eggs, respectively ( **Figure 1E** ). These data suggest that acute stress affects egg-laying behavior and/or fertility immediately and that this continues throughout the duration of stress-induced sleep.

To determine if egg-laying inhibition is reversible, we observed animals for 4 days following the same acute stress conditions. Heat-stressed animals showed reduced egg laying for 3 days compared to non-stressed controls, but on day 4 the animals displayed similar rates. UV-stressed animals did not recover and were sterile by day 4 ( **Figure 1C** ). These data suggest that heat-stressed animals undergo reversible egg reduction/retention, while UV-stressed animals have permanent defects in fertility.

We next sought to determine if egg retention in response to heat-stress is in part governed by behavior. To do this we examined the uterus of wild-type animals and reasoned that if behavior was a primary driver of the reduced egg laying, then animals would retain embryos of later ages. Animals were anesthetized and imaged using a 40X objective lens on a widefield microscope and total number of eggs and their ages was determined. Embryo development was categorized as previously described: 1) 1-8 cell, 2) 9-20 cell, 3) >21 cell, and 4) comma stage (Ringstad and Horvitz, 2008). No significant difference in the total number of eggs *in utero* after 8 hours between control and heat-stressed animals was observed ( **Figure 1D** ). However, we did find that control animals contained embryos primarily in early stages of development (1-8 or 9-20 cell stage), while heat-stressed animals contained more aged embryos. Specifically, the proportion of eggs in the 1-8 cell stage were significantly lower in heat-stressed animals, while 21+ cell stage embryos were observed more frequently ( **Figure 1E** ). Considering heat-stressed animals laid fewer eggs, but contained the same number as non-stressed controls, and that the embyros were aged, we conclude that egg laying inhibition during stress-induced sleep is a combination of behavioral quiescence and reduced fertility.

During waking, egg-laying is induced by serotonin from the HSN neurons, which activates vulval muscles (Waggoner et al. 1998). Ablation of the HSN neurons blocks egg laying, however, exogenous serotonin can bypass these effects (Desai and Horvitz, 1989). We reasoned that if inhibition of egg laying was in part behavioral then serotonin treatment would lead to more egg laying events. We found that serotonin did indeed increase egg laying over 8 hours in heat stressed animals, but not to the levels seen in non-stressed controls ( **Figure 1F** ). This further supports the notion that egg laying inhibition during stress-induced sleep is a combination of behavioral quiescence and reduced fertility.

Epidermal growth factor activation of the ALA and RIS neurons is required for quiescence of movement and feeding (Hill et al. 2014; Konietzka et al. 2020; Hill et al. 2024), and overexpression of *lin-3* , which encodes EGF ligands, induces movement, feeding, and defecation quiescence (Van Buskirk and Sternberg, 2007; Honer et al. 2020). To determine if egg laying can be modified by EGF signaling, we overexpressed *lin-3* from an inducible heat shock promoter using a mild heat stress of 33°C and measured egg laying 2 hours after heat exposure. We found that animals overexpressing *lin-3* laid significantly fewer eggs than both heat-shocked controls, and transgenic animals not heat shocked ( **Figure 1G** ). Thus, EGF signaling can reduce egg laying behavior.

Next, we tested the hypothesis that the ALA and/or RIS sleep neurons are required for egg laying inhibition during stress-induced sleep. We measured the number of eggs laid in 8 hours after heat- or UV-induced sleep, in *ceh-14* (ALA defective) (Van Buskirk and Sternberg, 2010) *, ceh-17* (ALA defective) (Pujol et al. 2000), *aptf-1* (RIS defective) (Turek et al. 2013), and *ceh-17* ; *aptf-1* (ALA and RIS defective) loss of function mutants. Prior work has demonstrated that the ALA is required for egg-laying suppression in response to intense mechanical-stimulation (Sanders et al. 2013), so we predicted that egg laying inhibition would be ALA-dependent. However, both the *ceh-14* and *ceh-17* mutants showed a strong reduction in egg laying during both heat- and UV-induced sleep ( **Figure 1H** ). These results suggest that the ALA alone is not required for the inhibition of egg laying. Next, we tested for the requirement of the RIS by measuring egg laying in *aptf-1* mutants, however, these animals also showed a significant reduction in eggs laid during both heat- and UV-induced sleep ( **Figure 1H** ). To rule out the possibility of redundancy of the ALA and RIS wemeasured egg laying in *ceh-17* ; *aptf-1* double mutants. Similar to our previous results, egg laying was significantly reduced during heat- and UV-induced sleep ( **Figure 1H** ), however, the percent change following heat stress in the double mutant was significantly lower than that of wild-type controls ( **Figure 1I** ). Overall, these data suggest that the ALA and RIS may be partially required for egg laying inhibition. To look at this further, we assessed the age of embryos in utero 8 hours after heat stress in *ceh-17* ; *aptf-1* mutants and found that the heat-stressed double mutants trended towards having younger embryos than heat-stressed wild-type animals. Specifically, they contained more 9-20 cell embryos and significantly fewer >21 cell embyros ( **Figure 1E** ). This suggests that the ALA and RIS are required for the full induction of egg laying behavioral quiescence during stress-induced sleep, but likely not involved with regulating fertility (i.e., egg production).

Last, we tested for the requirement of other neuropeptides known to regulate egg laying. The uv1 neuroendocrine cells, located in the uterus, play a significant role in mediating egg-laying activity (Collins et al. 2016; Banerjee et al. 2017). The uv1 cells secrete tyramine, and neuropeptides, including *nlp-7* and *flp-11* , that inhibit egg-laying activity (Collins et al. 2016; Banerjee et al. 2017). Additionally, the uv1 cells express the EGF receptor *let-23* (Chang et al. 1999), making the uv1 cells a potential target of EGF signaling during stress-induced sleep. As an initial test of this hypothesis, we measured egg laying in *nlp-7* and *flp-11* loss-of-function mutants. Like before, we found that both *nlp-7* and *flp-11* single, and *nlp-7* ; *flp-11* double mutants laid significantly fewer eggs during both heat- and UV-induced sleep. However, the double mutant showed a significant reduction in the overall percent change following heat stress ( **Figure 1I** ).

In summary, this study indicates that egg laying inhibition during stress-induced sleep is caused by a reduction in egg production, and behavioral quiescence of egg laying, however, the overall effects of behavior on the amount of progeny produced is small. This egg laying behavioral quiescence involves neuroendocrine signaling from the ALA , RIS, and uv1 cells.

## Methods

**Worm maintenance and strains**

Animals were maintained at 20°C on agar plates containing nematode growth medium and fed the OP50 derivative bacterial strain DA837 (Davis et al. 1995).

**Eight-hour egg laying assays**

L4 hermaphrodites were picked onto seeded plates the day prior to experiments. The next day, 3 adults were transferred onto seeded plates and subjected to one of the following: 1) no stress (controls); 2) heat-stress, where plates were sealed with parafilm and placed in a 37°C circulating water bath, for 30 minutes, and then animals were transferred to room-temperature seeded plates; and 3) UV-stress, plates with lids off were placed in a UV-cross linker (Stratagene) and exposed to 1500J/m ^2^ of UV irradiation. For each condition, the animals were allowed to lay eggs for an eight-hour period at 20°C. Following the eight-hour egg-laying period, animals were removed from the plates, a permanent marker was used to divide the plate into quadrants, and the number of eggs in each quadrant were counted. This was repeated over numerous trials and averages were calculated for each genotype, as well as the percent change between no stress and heat-stress for some genotypes. Averages and precent changes were compared by one-way ANOVA followed by Tukey's multiple comparisons test.

Egg-laying behavior was also evaluated hourly for the wild-type strain for the control, heat-stress, and UV-stress conditions. In these experiments, animals were moved onto new plates every hour for eight hours. Total eggs for each hour were quantified over multiple trials and compared across conditions by two-way ANOVA followed by Sidak's multiple comparisons test.

**Overexpression of *lin-3***

For transgenic animals capable of overexpressing *lin-3* using the inducible heat shock promoter, strain PS5009 (Van Buskirk and Sternberg 2007), only a mild heat shock of 33°C for 30 minutes was administered. L4 animals were transferred to seeded plates the day prior to all experiments, and day-one adults were heat shocked. Controls were allowed to lay eggs for a two-hour period at 20°C, with no manipulations, while plates containing heat-shocked animals were sealed with parafilm and placed in a 33°C circulating water bath for 30 minutes. The plates were then moved to a 20°C incubator for two hours to allow induction of the heat shock promoter, and then animals were transferred to seeded plates and allowed to lay eggs at 20°C for 2 hours. This was repeated over multiple trials, the average number of eggs laid was calculated, and compared between conditions and genotypes by one-way ANOVA followed by Tukey's multiple comparisons test.

**Serotonin egg laying assays**

To administer serotonin to the animals, 5-hydroxytryptamine (hydrochloride, Sigma) was mixed with DA837 bacteria to a final concentration of 25mM, and seeded onto standard growth plates. First day adults were transferred to the plates immediately prior to the 8-hour egg laying experiments.

## Reagents

The following strains were used in this study: N2 (Bristol - *wild type* ), PS5009 *pha-1* ( *e2132ts* ); *syEx723* [ *hsp-16.2p:* : *lin-3C* ; *myo-2p:gfp* ; *pha-1* ( *+* )], IB16 *ceh-17* ( *np1* )I, TB528 *ceh-4* ( *ch3* )X, NQ1065 *ceh-17* ( *np1* )I; *aptf-1* ( *gk794* )II, HBR227 *aptf-1* ( *gk794* )II, HBR507 *flp-11* ( *tm2706* )X, IZ1135 *nlp-7* ( *tm2984* )X, and IZ1589 *nlp-7* ( *tm2984* ); *flp-11* ( *tm2706* )X.

## References

[R1] Banerjee N, Bhattacharya R, Gorczyca M, Collins KM, Francis MM (2017). Local neuropeptide signaling modulates serotonergic transmission to shape the temporal organization of C. elegans egg-laying behavior.. PLoS Genet.

[R2] Chang C, Newman AP, Sternberg PW (1999). Reciprocal EGF signaling back to the uterus from the induced C. elegans vulva coordinates morphogenesis of epithelia.. Curr Biol.

[R3] Collins KM, Bode A, Fernandez RW, Tanis JE, Brewer JC, Creamer MS, Koelle MR (2016). Activity of the
*C. elegans*
egg-laying behavior circuit is controlled by competing activation and feedback inhibition.. Elife.

[R4] Davis MW, Somerville D, Lee RY, Lockery S, Avery L, Fambrough DM (1995). Mutations in the Caenorhabditis elegans Na,K-ATPase alpha-subunit gene, eat-6, disrupt excitable cell function.. J Neurosci.

[R5] DeBardeleben HK, Lopes LE, Nessel MP, Raizen DM (2017). Stress-Induced Sleep After Exposure to Ultraviolet Light Is Promoted by p53 in
*Caenorhabditis elegans*
.. Genetics.

[R6] Desai C, Horvitz HR (1989). Caenorhabditis elegans mutants defective in the functioning of the motor neurons responsible for egg laying.. Genetics.

[R7] Hill AJ, Mansfield R, Lopez JM, Raizen DM, Van Buskirk C (2014). Cellular stress induces a protective sleep-like state in C. elegans.. Curr Biol.

[R8] Hill AJ, Robinson B, Jones JG, Sternberg PW, Van Buskirk C (2024). Sleep drive is coupled to tissue damage via shedding of Caenorhabditis elegans EGFR ligand SISS-1.. Nat Commun.

[R9] Honer M, Buscemi K, Barrett N, Riazati N, Orlando G, Nelson MD (2020). Orcokinin neuropeptides regulate sleep in Caenorhabditis elegans.. J Neurogenet.

[R10] Iannacone MJ, Um P, Grubbs JI, van der Linden AM, Raizen DM (2024). Quiescence Enhances Survival during Viral Infection in Caenorhabditis elegans.. J Neurosci.

[R11] Konietzka J, Fritz M, Spiri S, McWhirter R, Leha A, Palumbos S, Costa WS, Oranth A, Gottschalk A, Miller DM 3rd, Hajnal A, Bringmann H (2019). Epidermal Growth Factor Signaling Promotes Sleep through a Combined Series and Parallel Neural Circuit.. Curr Biol.

[R12] McMullen PD, Aprison EZ, Winter PB, Amaral LA, Morimoto RI, Ruvinsky I (2012). Macro-level modeling of the response of C. elegans reproduction to chronic heat stress.. PLoS Comput Biol.

[R13] Mignerot L, Gimond C, Bolelli L, Bouleau C, Sandjak A, Boulin T, Braendle C (2024). Natural variation in the Caenorhabditis elegans egg-laying circuit modulates an intergenerational fitness trade-off.. Elife.

[R14] Nath RD, Chow ES, Wang H, Schwarz EM, Sternberg PW (2016). C.&nbsp;elegans Stress-Induced Sleep Emerges from the Collective Action of Multiple Neuropeptides.. Curr Biol.

[R15] Nelson MD, Lee KH, Churgin MA, Hill AJ, Van Buskirk C, Fang-Yen C, Raizen DM (2014). FMRFamide-like FLP-13 neuropeptides promote quiescence following heat stress in Caenorhabditis elegans.. Curr Biol.

[R16] Plagens RN, Mossiah I, Kim Guisbert KS, Guisbert E (2021). Chronic temperature stress inhibits reproduction and disrupts endocytosis via chaperone titration in Caenorhabditis elegans.. BMC Biol.

[R17] Prasanth MI, Santoshram GS, Bhaskar JP, Balamurugan K (2016). Ultraviolet-A triggers photoaging in model nematode Caenorhabditis elegans in a DAF-16 dependent pathway.. Age (Dordr).

[R18] Pujol N, Torregrossa P, Ewbank JJ, Brunet JF (2000). The homeodomain protein CePHOX2/CEH-17 controls antero-posterior axonal growth in C. elegans.. Development.

[R19] Ringstad N, Horvitz HR (2008). FMRFamide neuropeptides and acetylcholine synergistically inhibit egg-laying by C. elegans.. Nat Neurosci.

[R20] Sanders J, Nagy S, Fetterman G, Wright C, Treinin M, Biron D (2013). The Caenorhabditis elegans interneuron ALA is (also) a high-threshold mechanosensor.. BMC Neurosci.

[R21] Sawin ER. 1996. Genetic and cellular analysis of modulated behaviors in *Caenorhabditis elegans* , PhD thesis, Massachusetts Institute of Technology, Cambridge, Massachusetts

[R22] Schafer WR (2005). Egg-laying.. WormBook.

[R23] Sinner MP, Masurat F, Ewbank JJ, Pujol N, Bringmann H (2020). Innate Immunity Promotes Sleep through Epidermal Antimicrobial Peptides.. Curr Biol.

[R24] Turek M, Lewandrowski I, Bringmann H (2013). An AP2 transcription factor is required for a sleep-active neuron to induce sleep-like quiescence in C. elegans.. Curr Biol.

[R25] Van Buskirk C, Sternberg PW (2007). Epidermal growth factor signaling induces behavioral quiescence in Caenorhabditis elegans.. Nat Neurosci.

[R26] Van Buskirk C, Sternberg PW (2010). Paired and LIM class homeodomain proteins coordinate differentiation of the C. elegans ALA neuron.. Development.

[R27] Waggoner LE, Zhou GT, Schafer RW, Schafer WR (1998). Control of alternative behavioral states by serotonin in Caenorhabditis elegans.. Neuron.

